# 

**Published:** 1865-12

**Authors:** 


### CONTENTS.

ORIGINAL CONTRIBUTIONS.

ART. XXXIX. Report on the Means of Improving the Sanitary Condition of Chicago, 705

ART. XL. Typhoid Fever Complicated with Broncho-Pneumonia. Clinical Cases in the Medical Wards of Mercy Hospital. Substance of a Clinic. By N. S. Davis, M.D., Prof. Clinical Medicine, &c., 712

SELECTIONS.

On the Pathological Appearances Presented in Marsh Fever. By J. Forsyth Meigs, M.D., one of the Physicians to the Pennsylvania Hospital, 718

On the Use of Chloroform as an Internal Remedy. By A. P. Merrill, M.D., 727

Anæmia and Anæmiac Palpitation.—Physiology of the Blood.—Abstracts and Comments. By David Wooster, M.D., &c., 735

Sewers and their Evils, 739

BOOK NOTICES.

Researches on the Medicinal Properties and Applications of Nitrous Oxide, Protoxide of Nitrogen, or Laughing Gas. By Geo. J. Ziegler, M.D., 742

Materia Medica for the Use of Students. By John B. Biddle, M.D., 742

Stimulants and Narcotics, their Mutual Relations; with Special Researches on the Action of Alcohol, Æther, and Chloroform on the Vital Organism. By Francis E. Anstie, M.D., 743

Specialties in Medicine. By Henry D. Noyes, M.D., 743

EDITORIAL.

Wm. T. G. Morton and his Extraordinary Pretensions, 743

Death.—Dr. Ezra A. Steele, 752

Army Surgeons Returned, 752

Something Wrong.—Surgeon-General, 752

Longevity of Classes, 753

The New Orleans School of Medicine, 753

Deaths.—Timothy Childs, M.D., Sir William Hooker, 753

The Mortality Statistics for October, 1865, 754

Revival of Medical Literature and Medical Schools in the South, 755

Military Surgery in France, 755

Antagonistic Effects of Calabar Bean and Atropia, 756

Entozoa in the Calf, 757

Diphtheria.—A Circular from Dr. Norton, 758

Dry Gangrene of the Hand from Injections into an Aneurismal Sac, 758

Appliances for Invalids, 759

Singular Electrical Phenomena in the Human Body following Lightning Stroke, 759

Danger of Subcutaneous Injections, 760

Injection of Air into Foul Abscesses and Fistulæ, 760

Treatment of Choryza, 761

Records of the Medical Department, 761

A Fossil Man dug up between Veyziat and Oyonnax, near Nantua, (Ain), 761

Longevity in England and Wales, 761

Prof. Malgaigne, 762

A Prize on the Vaccino-Syphilitic Question, 762

London Lancet, 762

New Medical School at Cairo, Illinois, 726

